# Prevalence estimates of tuberculosis infection in adults in Denmark: a retrospective nationwide register-based cross-sectional study, 2010 to 2018

**DOI:** 10.2807/1560-7917.ES.2024.29.12.2300590

**Published:** 2024-03-21

**Authors:** Anne Ahrens Østergaard, Troels Lillebaek, Inge Petersen, Andreas Fløe, Eliza H Worren Bøkan, Ole Hilberg, Inge K Holden, Lone Larsen, Ada Colic, Christian Wejse, Pernille Ravn, Bente Mertz Nørgård, Stephanie Bjerrum, Isik Somuncu Johansen

**Affiliations:** 1 Department of Infectious Diseases and Mycobacterial Centre for Research Southern Denmark, MyCRESD, Odense University Hospital, Denmark; 2 Research Unit of Infectious Diseases, Department of Clinical Research, University of Southern Denmark; 3 International Reference Laboratory of Mycobacteriology, Statens Serum Institut, Denmark; 4 Department of Public Health, University of Copenhagen, Denmark; 5 Department of Respiratory Diseases and Allergy, Aarhus University Hospital, Aarhus, Denmark; 6 Department of Medicine, Vejle Hospital, Hospital Lillebælt, Vejle, Denmark; 7 Department of Gastroenterology and Hepatology, Aalborg University Hospital, Aalborg, Denmark; 8 Center for Molecular Prediction of Inflammatory Bowel Disease, PREDICT, Department of Clinical Medicine, The Faculty of Medicine, Aalborg University, Denmark; 9 Department of Rheumatology, Zealand University Hospital, Køge, Denmark; 10 Department of Infectious Diseases, Aarhus University Hospital, Aarhus, Denmark; 11 GloHAU, Center for Global Health, Department of Public Health, Aarhus University, Aarhus, Denmark; 12 Section for Infectious Diseases, Department of Medicine, Herlev and Gentofte Hospital, Copenhagen, University of Copenhagen, Gentofte, Denmark; 13 Center of Clinical Epidemiology, Odense University Hospital and Research Unit of Clinical Epidemiology, University of Southern Denmark, Odense, Denmark; 14 Department of Infectious Diseases, Copenhagen University Hospital, Rigshospitalet, Copenhagen, Denmark

**Keywords:** Latent tuberculosis infection, Screening, Interferon Gamma Release Assay, Prevalence, Sample weighting

## Abstract

**Background:**

Tuberculosis (TB) elimination requires identifying and treating persons with TB infection (TBI).

**Aim:**

We estimate the prevalence of positive interferon gamma release assay (IGRA) tests (including TB) and TBI (excluding TB) in Denmark based on TBI screening data from patients with inflammatory bowel disease (IBD) or inflammatory rheumatic disease (IRD).

**Methods:**

Using nationwide Danish registries, we included all patients with IBD or IRD with an IGRA test performed between 2010 and 2018. We estimated the prevalence of TBI and positive IGRA with 95% confidence intervals (CI) in adolescents and adults aged 15–64 years after sample weighting adjusting for distortions in the sample from the background population of Denmark for sex, age group and TB incidence rates (IR) in country of birth.

**Results:**

In 13,574 patients with IBD or IRD, 12,892 IGRA tests (95.0%) were negative, 461 (3.4%) were positive and 221 (1.6%) were indeterminate, resulting in a weighted TBI prevalence of 3.2% (95% CI: 2.9–3.5) and weighted positive IGRA prevalence of 3.8% (95% CI: 3.5–4.2) among adults aged 15–64 years in the background population of Denmark. Unweighted TBI prevalence increased with age and birthplace in countries with a TB IR higher than 10/100,000 population.

**Conclusion:**

Estimated TBI prevalence is low in Denmark. We estimate that 200,000 persons have TBI and thus are at risk of developing TB. Screening for TBI and preventive treatment, especially in persons born in high TB incidence countries or immunosuppressed, are crucial to reduce the risk of and eliminate TB.

**Key public health message**

**What did you want to address in this study and why?**

We wanted to estimate the prevalence of tuberculosis infection in people aged between 15 and 64 years in Denmark. Tuberculosis infection refers to infection with *Mycobacterium tuberculosis* without disease. Tuberculosis is a disease that can be eliminated and the World Health Organization aims to achieve this by 2035. To accomplish this goal, it is necessary to identify and treat people with tuberculosis infection, since they represent a potential reservoir of tuberculosis.

**What have we learnt from this study?**

The prevalence of tuberculosis infection in adolescents and adults Denmark is 3.2%, corresponding to ca 200,000 people infected with the tuberculosis bacterium. This suggests a reservoir of 10,000 to 20,000 potential tuberculosis cases in the future. The prevalence of tuberculosis infection increases with age and the incidence rate in the country of birth.

**What are the implications of your findings for public health?**

Systematic screening for tuberculosis infection and preventive treatment should be implemented for people with immunosuppression or from countries with a tuberculosis incidence rate greater than 10 per 100,000 population.

## Introduction

Management of (latent) tuberculosis infection (TBI) is an important component in the tuberculosis (TB) elimination strategy in low TB incidence countries, according to the World Health Organization (WHO) action plan [1]. Thus, screening for TBI and treating persons with known risk factors of progression to the active disease is crucial, particularly due to the increasing use of immunosuppressive biological agents and migrants arriving from high TB incidence countries [2].

Tuberculosis infection can be defined as a persistent immune response to stimulation by *Mycobacterium tuberculosis* antigens with no evidence of active TB disease. However, no specific standard for diagnosing TBI exists. How a TB survivor with a persistent immune response to stimulation by *M. tuberculosis* antigens classifies is not well defined. A quarter of the world’s population has TBI and their lifetime risk of progression to TB without TB preventive treatment (TPT) is ca 10%, depending on the individual risk factors [3,4].

The latest European tuberculosis surveillance report revealed a 1.2% increase in TB incidence since 2021, increasing for the first time in two decades [5]. In Denmark, TB decreased during the last two decades to an incidence of 3.6 per 100,000 population in 2021 [6]. However, the decline has stagnated in the past 3 years. Additionally, the prevalence of TBI in Denmark is unknown because no systematic screening studies have been performed. The prevalence of TBI has been estimated to be 3–5% based on TB incidence rates (IR) in Denmark in 2019 [7]. Still, much higher TBI prevalence (8–17%) has been documented among socially marginalised people, immigrants and refugees from high TB incidence areas [8,9]. To reduce the TBI reservoir, it is necessary to determine the national prevalence of TBI and identify specific populations of interest for targeted interventions. However, Denmark does not yet have a programme in place for TB elimination.

Patients treated with immunosuppressive biological agents are at particularly high risk of progression from TBI to active TB [10]. Therefore, since 2009, Danish guidelines have recommended systematic screening by interferon gamma release assays (IGRA) for TBI regardless of exposure to *M. tuberculosis* before initiation of biological agents to treat inflammatory bowel disease (IBD) and rheumatic disease (IRD) [11]. In 2009, the prevalence of TBI was estimated to be 0–7% using IGRA tests among patients with IBD and IRD in Denmark [12].

We aim to estimate the prevalence of TBI and positive IGRA tests, including positive IGRA tests of persons diagnosed with TB, based on the TBI screening data from IGRA-tested patients with IBD or IRD in Denmark between 2010 and 2018.

## Methods

This nationwide retrospective register-based period cross-sectional study estimates the prevalence of TBI and positive IGRA tests in Denmark.

**Figure 1:**
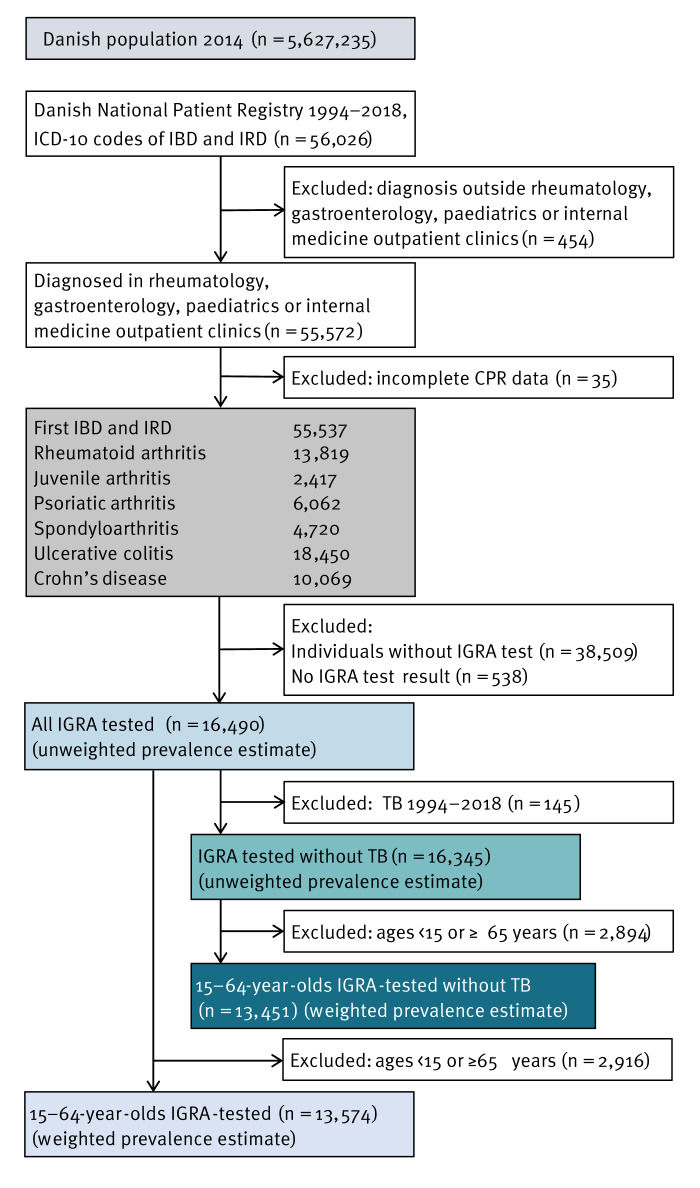
Total population of Denmark in 2014, number of patients from the Danish National Patient Registry with diagnoses of rheumatic disease or inflammatory bowel disease between 1994 and 2018 and interferon gamma release assay results, 2010–2018

CPR: Danish civil registration system; IBD: Inflammatory bowel disease; ICD10: International Classification of Diseases 10th Revision; IGRA: interferon gamma release assay; IRD: inflammatory rheumatic disease; TB: tuberculosis.

Patients diagnosed with either an IRD (rheumatoid arthritis, juvenile arthritis, psoriatic arthritis or spondyloarthritis) or IBD (Crohn’s disease or ulcerative colitis) in a gastroenterology, rheumatology, internal medicine or paediatric outpatient clinic (Supplementary Table S1) were identified in the Danish National Patient Registry (DNPR) between 1994 and 31 December 2018 (Figure 1). The DNPR contains all International Classification of Diseases 10th Revision (ICD-10) diagnosis codes at hospital admission and for outpatient contacts from Danish public hospitals since 1994 [13]. From the DNPR, we obtained information on the TB diagnosis status of patients with IBD or IRD (Supplementary Table S1) between 1994 and 2018 and data on which patients received treatment with biological agents (procedure code BOHJ18) between 2010 and 2018. Inflammatory diagnoses were sorted arbitrarily in case of multiple diagnoses on the first date a diagnosis code was assigned.

From the Danish Civil Registration (CPR) system, which contains high-accuracy data on all citizens living in Denmark [14], we obtained date of birth, sex and country of birth.

We cross-linked register data by the unique 10-digit personal identifier (CPR number) assigned to all residents at birth or after residing legally in Denmark for 3 months. A few persons with an invalid CPR number could not be cross-linked and were excluded from all analyses.

From the Danish Microbiology Database (MiBa), which contains microbiological test results from all Danish microbiology laboratories since 2010, we obtained data on microscopy (acid-fast bacilli), mycobacterial culture and specific PCR tests for the *M. tuberculosis* complex between 2010 and 2018 to identify patients with microbiologically-verified TB.

From MiBa, we also obtained IGRA results (QuantiFERONTB*-*Gold and QuantiFERON TB*-*Gold Plus, QIAGEN Benelux, Venlo, the Netherlands) for patients diagnosed with IBD or IRD between 2010 and 2018. According to the manufacturer’s instructions, the laboratory reported the IGRA results as positive, negative or indeterminate [15].

We supplemented the MiBa data with locally performed *T-SPOT* TB tests (Oxford Immunotec, Oxford, United Kingdom) at the Department of Respiratory Medicine, Aarhus University Hospital between January 2010 and May 2018, and data on laboratory test results from three hospitals from the Danish National Laboratory Registry [13].

Only one sample per patient per day was included. In the case of multiple tests, we used any positive test as an IGRA result. If the first test was indeterminate, a later negative or positive test was used as the IGRA result.

Patients with IBD and IRD should be screened for TBI if the clinician considers initiating biological therapy [11]. It is recommended to use a tuberculin skin test (TST), IGRA or T-spot. However, it is not mandatory for those who do not initiate biological therapy. Since TBI screening is performed before biological therapy regardless of TB exposure, this tested population provides a suitable sample for estimating the prevalence of TBI in the background population.

We excluded all persons without IGRA test results. A TBI diagnosis was defined as a positive IGRA without TB. A TB diagnosis was based on an ICD-10 diagnosis of TB or a verified positive microbiological test for TB between 2010 and 2018 using data from MiBa. In our estimate of TBI, we excluded the IGRA results of patients diagnosed with TB between 1994 and 2018 to ensure that the IGRA result was not positive due to incipient TB.

### Background population

Demographics (sex, age during the mid-study period and country of birth) used for establishing sample weights and region of residence of the background population in Denmark on 1 January 2014, were retrieved from an openly accessible database [16]. We chose January 2014 as the mid-point between 2010 and 2018.

### Outcomes

The primary outcome variables were the prevalence of TBI and a positive IGRA test. The secondary outcome variables were the demographical characteristics (sex, age group and TB IR by country of birth) of patients with IBD or IRD, and TBI.

### Statistical analysis

For both the study sample of patients with IBD or IRD and the background population, we calculated distributions of sex, age group (15–44 and 45–64 years) and IR of TB in the country of birth (low < 10 TB cases/100,000 population; medium 10–40/100,000; high > 40/100,000, see Supplementary Table S2 for weights and Supplementary Table S3 for classification according to the WHO).

We used sampling weights to generate a nationally representative estimate of TBI prevalence and positive IGRA. Sample weighting is a method of weighting groups to represent the background population as though the study sample were a random draw of the total population [17]. We thereby adjusted for distortion in the study sample for sex, age group and TB IR in the country of birth compared with the background population. To ensure a reasonable size of weights, we excluded persons younger than 15 and older than 64 years old. This was due to a small number of individuals in this age range with TBI and born in a country with medium or high TB IR (n < 6).

The 9-year period prevalence from 2010 to 2018 and 95% confidence intervals (CI) of TBI and positive IGRA in age, sex and TB IR classification of the country of birth were estimated for all ages and also for age 15–64 years (unweighted estimate). We used sample weights (Stata command: fweight) to estimate the prevalence of TBI and positive IGRA in the background population of Denmark aged 15–64 years (weighted estimate). In the prevalence calculation, a non-positive IGRA result (negative or indeterminate) is considered a negative result. Data were analysed using Stata/BE version 17.0 (StataCorp, College Station, United States (US)).

## Results

There were 5,627,235 inhabitants in Denmark on 1 January 2014, the mid-study period. After excluding persons with incomplete CPR data (n = 35), 55,537 persons had a diagnosis of IBD or IRD from a specialised department between 2010 and 2018 (Figure 1). We further excluded persons without an IGRA test (n = 38,509) or with no definite IGRA test result (n = 538), leaving 16,490 patients for the subsequent analysis, corresponding to 0.29% of the background population (Table 1). The median mid-period age in all IGRA-tested persons with IBD or IRD was 42.1 (interquartile range (IQR): 27.9–56.3) years compared with 41 (IQR: 21–59) years in the background population. Of all IGRA-tested persons, 15,016 (91.1%) originated from Denmark, compared with 89.9% in the background population. Most IGRA-tested persons were female (56.9%) and born in countries with low TB IR (93.9%). In the background population, 50.4% were female and 92.7% were born in a low TB IR country. The background population consisted of 17.2% aged 0–14 years, 18.2% aged 15–44 years, 26.6% aged 45–64 years and 37.9% older than 65 years. Among persons tested with IGRA, 5.5% were aged 0–14 years, 49.3% were aged 15–44, 33.0% were aged 45–64 and 12.2% were aged 65 or older. There was an equal distribution of IGRA testing throughout the country (Supplementary Table S4 displays the percentages of IGRA tests conducted in the five Danish regions).

**Table 1:** Demographics of the background population, persons with inflammatory bowel disease or rheumatic disease, all interferon gamma release assay tested persons, including persons diagnosed with tuberculosis and excluding persons diagnosed with tuberculosis, Denmark, 2010–2018

	Background population of Denmark mid-study period, 2014		Persons with IBD or IRD		Persons with IBD or IRD tested with IGRA, including those with TB		Persons with IBD or IRD tested with IGRA, excluding those with TB	
	**n**	**%**	**n**	**%**	**n**	**%**	**n**	**%**
Total	5,627,235	100.0	55,537	0.99	16,490	0.29	13,451	0.24
Median age (IQR), mid-study period in years^a^	41 (21–59)		49.4 (34.9–62.6)		42.1 (27.9–56.3)		40.3 (28.4–51.8)	
Age group in 2014								
0–14 years	968,670	17.2	1,701	3.1	900	5.5	NA	NA
15–44 years	1,026,734	18.2	21,376	38.5	8,131	49.3	8,073	40.0
45–64 years	1,497,949	26.6	20,742	37.3	5,443	33.0	5,378	60.0
≥ 65 years	2,133,882	37.9	11,718	21.1	2,016	12.2	NA	NA
Sex (female)	2,836,126	50.4	32,267	58.1	9,383	56.9	7,546	56.1
Birthplace								
Denmark	5,056,810	89.9	51,339	92.4	15,016	91.1	12,166	90.4
Outside Denmark	570,425	10.1	4,198	7.6	1,474	8.9	1,285	9.6
TB IR in country of birth								
Low (< 10/100,000)	5,216,625	92.7	52,882	95.2	15,484	93.9	12,565	93.4
Medium (10–40/100,000)	180,688	3.2	1,350	2.4	498	3.0	448	3.3
High (> 40/100,000)	229,922	4.1	1,305	2.3	508	3.1	438	3.3

IBD: inflammatory bowel disease; IGRA: interferon gamma release assay; IQR: interquartile range; IR: incidence rate; IRD: inflammatory rheumatic disease; NA: not applicable; TB: tuberculosis.

^a^ Background data population were obtained in integers, and no decimals can be provided.

Of 16,490 IGRA tests, 15,659 (95.0%) were negative, 543 (3.3%) were positive and 288 (1.7%) were indeterminate (Table 2), resulting in a prevalence of positive IGRA at 3.3% (95% CI: 3.0–3.6). Of 13,474 IGRA tests in people aged 15–64 years, including persons diagnosed with TB, 12,892 (95.0%) were negative, 461 (3.4%) were positive and 221 (1.6%) were indeterminate. When sample weighting for the distribution of sex, age group and TB IR in the country of birth, we estimated the prevalence of a positive IGRA to be 3.8% (95% CI: 3.5–4.2) in the population of Denmark aged 15–64 years. Among 16,345 IGRA-tested persons without TB, 446 (2.6%) had a positive IGRA, resulting in a TBI prevalence of 2.7% (95% CI: 2.5–3.0). In adults aged 15–64 years, excluding persons diagnosed with TB, 376 of 13,451 (2.8%) had a positive IGRA test, resulting in a weighted prevalence of TBI for the population of Denmark aged 15–64 years of 3.2% (95% CI: 2.9–3.5).

**Table 2:** Interferon gamma release assay results of all tested and exclusive persons diagnosed with tuberculosis, and period prevalence estimates of tuberculosis infection and positive tests, Denmark, 2010–2018 (n = 16,490)

Group	All IGRA tested (inclusive persons diagnosed with TB 1994–2018)				IGRA tested (exclusive persons diagnosed with TB 1994–2018)			
Age group	All		15–64 years		All		15–64 years	
	n	%	n	%	n	%	n	%
Total	16,490	100.0	13,574	100.0	16,345	100.0	13,451	100.0
IGRA result								
Negative	15,659	95.0	12,892	95.0	15,616	95.5	12,857	95.6
Positive	543	3.3	461	3.4	446	2.6	376	2.8
Indeterminate	288	1.7	221	1.6	283	1.7	218	1.6
Period prevalence	Positive IGRA				TBI			
	%	95% CI	%	95% CI	%	95% CI	%	95% CI
Unweighted^a^ estimate	3.3	3.0–3.6	3.4	3.1–3.7	2.7	2.5–3.0	2.8	2.5–3.1
Weighted^b^ estimate	NA	NA	3.8	3.5–4.2	NA	NA	3.2	2.9–3.5

CI: confidence interval; IGRA: interferon gamma release assay; NA: not applicable; TB: tuberculosis; TBI: tuberculosis infection.

^a^ Unweighted estimates apply to the sample.

^b^ Weighted estimates apply to the background population of Denmark. Due to few persons with TBI in ages younger than 15 and older than 64 years born in countries with medium and high TB incidence rates, we excluded these age groups to ensure reasonable size of weights.

In the excluded persons younger than 15 years and older than 64 years, the unweighted prevalence of TBI among persons in the study younger than 15 years and older than 64 years was 1.1% (95% CI: 0.4–1.8) and 3.0% (95% CI: 2.3–3.8), respectively (data not shown).

**Figure 2:**
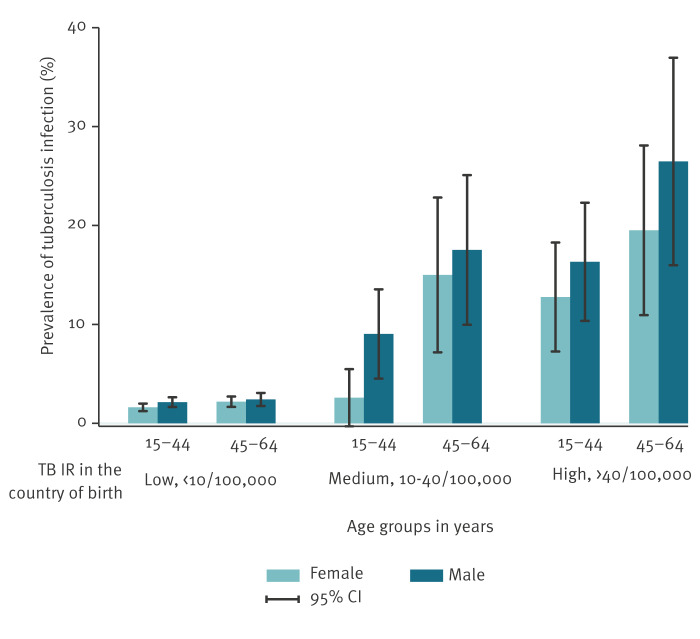
The prevalence of tuberculosis infection, unweighted, in interferon gamma release assay-tested people aged 15–64 years without persons diagnosed with tuberculosis by sex, age group, and tuberculosis incidence rate in country of birth, Denmark, 2010–2018 (n = 376)

CI: confidence interval; IR: incidence rate; TB: tuberculosis.

The unweighted prevalence of TBI tended to increase with age (Figure 2). A higher unweighted prevalence of TBI was observed in persons born in countries with middle and high TB IR. All groups had a higher prevalence of TBI for males than females.

## Discussion

In this study, we estimated the weighted 9-year period prevalence of TBI in adolescents and adults aged 15–64 years in Denmark. The estimated weighted prevalence of TBI in this age group was 3.2%, and 3.8% for a positive IGRA. The unweighted TBI prevalence increased with age, markedly for persons born in countries with TB IR greater than 10 per 100,000 population.

This study presents estimates of TBI prevalence and identifies specific populations of interest for targeted interventions in Denmark. Although Denmark does not yet have a programme in place for TB elimination, our estimates are valuable in supporting the TBI cascade of care and guiding national policymakers in Denmark and other European countries with similar TB epidemiology.

We estimated the prevalence of both TBI and a positive IGRA, including persons with a TB diagnosis before or after IGRA testing, since persons with previous TB have an increased risk of recurrent TB [18]. All IGRA-tested persons, including those diagnosed with TB between 1994 and 2018, had a slightly higher percentage of positive IGRA tests resulting in a slightly higher estimated prevalence of a positive IGRA than the prevalence of TBI.

Our estimate of TBI corresponds to potentially 200,000 persons with TBI in Denmark in 2014. Based on TB progression risk of 5–10%, this represents a possibility of 10,000 to 20,000 persons falling ill from TB in their remaining lifetime. The population of both Denmark and the world is ageing [19], and TB prevalence and mortality increase with age [20,21] due to an alternated immune response to TBI [22] and the possible influence of increased comorbidities. The risk of progression from TBI to TB is highest in the first few years of infection [23]. However, the risk of progression to TB may also increase after the age of 65 years, but the increased TBI prevalence’s contribution to the overall risk of TB progression is unclear and the increase could be due to a new infection [24]. We did not adjust for the duration of TBI or alternating risk of progression to TB. Denmark has not yet established national data on TPT. Nevertheless, the national guidelines recommend TPT for people with immunosuppression [11].

In Denmark, non-Danish origin was recorded for 76% of persons diagnosed with new TB in 2021, and an estimated 74% were infected outside Denmark based on notification data reported during patient interviews [6]. The incidence of TB is highest during the first year following arrival in Denmark (275/100,000 person-years) and remains higher for more than a decade compared with the population born in Denmark [2]. Accordingly, the Danish Health and Medicines Authority recommends screening people seeking asylum in Denmark from high TB incidence countries [25]. Still, is not regularly performed and TPT is not always offered in Denmark since data show that the number of persons offered TPT is low [9,25,26]. However, an improved cascade of care through a community-engaged approach can improve screening uptake and treatment completion, as Spruijt et al. showed [27].

Our results show that persons aged 45–64 years may have a higher prevalence of TBI compared to persons aged 15–44 years based on TST and annual risk of infection in the country of birth [28]. One study reported an increase in TBI prevalence by IGRA in persons aged 45-64 years compared to persons aged 15–44 years, but a decrease in in TBI prevalence by TST in adults older than 65 years [29]. Increased TBI prevalence in older adults could be due to previously higher TB incidence rates globally [30]. On looking at IGRA results only, our data suggest that the prevalence of TBI increases with age. This could be due to the better performance of IGRA tests than TST in patients with inflammatory diseases and older adults [31].

Our findings coincide with the TBI prevalence estimates by Cohen et al. [7] using extrapolation. In the US, between 2014 and 2017, TBI prevalence was estimated to be 2.7% [32]. The study used a back-calculation method of previous TB reactivation rates after weighting for population size, age group, race/ethnicity and country of birth. Likewise, they found an increase in the prevalence of TBI with increasing age. In Australia in 2016, the prevalence of TBI was estimated to be 5.1% [28]. Australia has higher migration levels than Denmark, thus a possible explanation for higher TBI prevalence is a greater number of migrants from high TB incidence countries in Australia compared with Denmark [33].

We found that 1.7% of all IGRA-tested persons had an indeterminate result. This is lower than previous findings of 5% in persons with immune-mediated inflammatory diseases in Denmark and internationally [12,34]. We allowed for re-testing, which may explain the low percentage of indeterminate results. Danish data of all IGRA-tested persons found that no one with an indeterminate result developed TB within 2 years after IGRA testing [35]. European data of 199 patients with autoimmune diseases found higher percentages with indeterminate IGRA results, but none progressed to TB in the median follow-up of 2.4 years [36]. These findings support that although indeterminate results were included as negatives, we did not underestimate TBI prevalence.

The strengths of this study are that estimates of TBI prevalence were based on testing in patients with IBD or IRD. This minimised confounding by test indication since we consider this group representative of the background population regarding the risk of TBI, as supported by an equal distribution of IGRA testing throughout the country. In addition, including data on TB incidence in country of birth allows for a more nuanced picture of TBI prevalence, and the weighting of the background population distribution of demographics, diagnostics and diagnoses based on nationwide Danish registers allows robust estimates for a complete national sample.

Limitations of this study were that data did not include persons diagnosed with TBI in all age groups, sex and TB IR in the country of birth. Hence, we only estimated TBI prevalence in the background population of Denmark aged between 15 and 64 years. Diagnosis of TBI lacks a gold standard and may not be complete when relying solely on IGRA results [37]. Additionally, low quantitative interferon gamma response in patients with inflammatory disease may have affected IGRA results, leading to false negatives [38]. All of these situations could lead to an underestimation of TBI prevalence. Our data did not include information on TPT. Our estimate applies to the background population of Denmark, which is not routinely screened and therefore not treated. Consequently, TPT had little impact on the prevalence estimate. Another limitation is that Departments of clinical immunology may have analysed a smaller number of IGRAs locally during the study period and these data may not be included.

## Conclusion

In conclusion, although the prevalence of TBI in Demark is low (3.2%) in this study, data suggest considerable differences in TBI prevalence depending on TBI prevalence in the country of birth. We estimate that 200,000 residents of Denmark have TBI and are potentially at risk of developing TB. To achieve TB elimination in Denmark by 2035, targeted testing and preventive treatment in immunosuppressed patients and populations from high TB incidence countries is crucial.

## Supplementary Data

265000 Supplementary Material
