# Supplementary material for: Prevalence estimates of tuberculosis infection in adults in Denmark: a retrospective nationwide register-based cross-sectional study, 2010 to 2018
Source: Euro Surveill. 2024 Mar 21;29(12):2300590. doi: 10.2807/1560-7917.ES.2024.29.12.2300590 (PMC11063675; doi:10.2807/1560-7917.ES.2024.29.12.2300590)
Supplement: Supplementary Material [file 23-00590_OSTERGAARD_Supplement.pdf]

This supplementary material is hosted by *Eurosurveillance* as supporting information alongside the article 'Prevalence estimates of tuberculosis infection in adults in Denmark: a retrospective nationwide register-based cross-sectional study 2010 to 2018', on behalf of the authors, who remain responsible for the accuracy and appropriateness of the content. The same standards for ethics, copyright, attributions and permissions as for the article apply. Supplements are not edited by *Eurosurveillance* and the journal is not responsible for the maintenance of any links or email addresses provided therein.

Table S1. International Classification of Diseases 10th Revision codes for inflammatory bowel diseases, inflammatory rheumatic diseases, and tuberculosis obtained from the Danish National Patient Registry, 1994-2018.

Disease	International Classification of Diseases 10th Revision codes
**Crohn's disease**	DK50, DM074, DK500, DK500A, DK500B, DK500C, DK501, DK501D, DK508, DK508A, DK508C, DK508D, DK509, DO996A
**Ulcerative Colitis**	DK51, DK510, DK512, DK513, DK514, DK515, DK515A, DK515B, DK518, DK518B, DK519
**Rheumatoid Arthritis**	DM05, DM050, DM051, DM051A, DM051B. DM051C, DM051D, DM051E, DM051F, DM052, DM053, DM058, DM059
**Juvenile Arthritis**	DM08, DM080, DM080A, DM080B, DM081, DM082, DM082A, DM082B, DM083, DM084, DM088, DM088A, DM089, DM09, DM090, DM091, DM092, DM098
**Spondyloarthritis**	DM45, DM459, DM46, DM460, DM468, DM468A, DM469
**Psoriatic arthritis**	DM07, DM070, DM071, DM072, DM073, DM073A, DM073B, DM074, DM075, DM076
**Tuberculosis**	DA15, DA16, DB909, DA17, DA18, DA19, DK930, DM011, DM900, DN330, DN741, DN741A, DO980, DP370

Table S2. Sample weighting after number and percentage of the background population, all Interferon Gamma Release Assay tested including tuberculosis cases, and positive Interferon Gamma Release Assay tests by sex, age groups (15-64-year-olds), and tuberculosis incidence rates in the country of birth in Denmark, 2010-2018.

**Sex**	**Age group** in years	**TB IR in the country of birth**	**Background population 2014**		**All IGRA-tested incl. TB,** n (%)		**Positive IGRA test** (inclusive tuberculosis cases)	
			n	%	n	%	n	%
Female	15-44	Low <10/100,000	924,081	16.4	4,174	25.3	80	14.7
Male	15-44	Low <10/100,000	958,535	17.0	3,375	20.5	84	15.5
Female	45-64	Low <10/100,000	693,399	12.3	3,002	18.2	84	15.5
Male	45-64	Low <10/100,000	696,316	12.4	2,084	12.6	54	9.9
Female	15-44	Medium 10-40/100,000	48,654	0.9	120	0.7	5	0.9
Male	15-44	Medium 10-40/100,000	54,087	1.0	159	1.0	17	3.1
Female	45-64	Medium 10-40/100,000	23,944	0.4	82	0.5	14	2.6
Male	45-64	Medium 10-40/100,000	31,155	0.6	105	0.6	21	3.9
Female	15-44	High >40/100,000	81,709	1.5	145	0.9	20	3.7
Male	15-44	High >40/100,000	66,816	1.2	158	1.0	32	5.9
Female	45-64	High >40/100,000	30,104	0.5	89	0.5	21	3.9
Male	45-64	High >40/100,000	23,031	0.4	81	0.5	29	5.3
Total adults aged 15-64 years:			3,631,831	64.5	13,574	82.3	461	84.9

IGRA: Interferon Gamma Release Assay. IR: incidence rate. TB: tuberculosis.

Table S3. Tuberculosis incidence rate estimates mid-cohort 2014 by birth countries included.

TB incidence rate category	Country	TB incidence rate /100,000
**High** >40/ 100,000	Afghanistan	189
	Algeria	72
	Angola	370
	Armenia	57
	Azerbaijan	76
	Bangladesh	221
	Belarus	51
	Benin	61
	Bhutan	181
	Bolivia	120
	Bosnia and Herzegovina	43
	Botswana	385
	Burkina Faso	54
	Burundi	126
	Brazil	43
	Brunei Darussalam	55
	Cabo Verde	66
	Cambodia	383
	Cameroon	220
	Chad	145
	Central African Republic	540
	Congo, Democratic Republic	325
	Congo, Republic	381
	Côte d'Ivoire	165
	China	67
	Djibouti	337
	Dominican Republic	54
	Ecuador	40
	El Salvador	44
	Equatorial Guinea	260
	Eritrea	119
	Eswatini	855
	Ethiopia	207
	Gabon	541
	Gambia, The	175
	Georgia	106
	Ghana	165
	Guinea	177
	Guinea-Bissau	361
	Greenland	179
	Guyana	89
	Haiti	200

	Hong Kong Special Administrative Region, China	74
	India	263
	Indonesia	329
	Kazakhstan	91
	Kiribati	473
	Kyrgyz Republic	126
	Kenya	423
	Korea, Democratic People's Republic	513
	Korea, Republic	85
	Kosovo	44[b]
	Lao PDR	189
	Liberia	308
	Latin America & Caribbean (excluding high income)	41
	Latvia	42
	Lesotho	853
	Lithuania	57
	Madagascar	233
	Malawi	228
	Malaysia	93
	Mail	58
	Marshall Islands	310
	Mongolia	428
	Morocco	101
	Mauritania	111
	Moldova	115
	Mozambique	361
	Myanmar	411
	Namibia	674
	Nauru	84
	Nepal	276
	Nigeria	219
	Niger	98
	Nicaragua	54
	Pakistan	270
	Panama	47
	Papua New Guinea	432
	Peru	125
	Philippines	546
	Romania	85
	Russian Federation	70
	Rwanda	65
	Sao Tome and Principe	141
	Senegal	125
	Sierra Leone	310
	Singapore	45
	Solomon Islands	72
	Somalia	274
	South Africa	1170

	South Sudan	227
	Sri Lanka	65
	Sudan	94
	Taiwan	[a]
	Tanzania	327
	Thailand	167
	Tajikistan	91
	Timor-Leste	498
	Togo	58
	Turkmenistan	58
	Yemen, Republic	48
	Uganda	202
	Ukraine	94
	Uzbekistan	82
	Vanuatu	63
	Vietnam	205
	Zambia	406
	Zimbabwe	278
**Medium** 10-40/ 100,000	Albania	16
	Argentina	25
	Bahamas, the	15
	Bahrain	17
	Belize	23
	Bulgaria	31
	Chile	15
	Colombia	31
	Comoros	35
	Costa Rica	12
	Croatia	13
	Egypt, Arab Republic	15
	Europe & Central Asia	35
	Estonia	21
	Guatemala	25
	Honduras	39
	Iran, Islamic Republic	16
	Iraq	39
	Japan	18
	Kuwait	22
	Lebanon	12
	Libya	40
	Maldives	36
	Malta	12
	Mauritius	13
	Mexico	22
	Middle East & North Africa	36
	Montenegro	21
	North Macedonia	17
	Oman	10

	Paraguay	39
	Poland	19
	Portugal	24
	Qatar	24
	Samoa	13
	Saudi Arabia	12
	Serbia	23
	Seychelles	15
	Spain	12
	Suriname	33
	Syrian Arab Republic	23
	Tonga	14
	Trinidad and Tobago	20
	Tunisia	35
	Turkiye	20
	United Kingdom including Falkland Islands	12
	Uruguay	29
	Venezuela	27
**Low** < 10/ 100,000	Andorra	10
	Aruba	2
	Australia	7
	Antigua and Barbuda	4
	Austria	8
	Barbados	2
	Belgium	9
	Bermuda	0
	British Virgin Islands	0
	Canada	5
	Cuba	7
	Curacao	3
	Cypress	4
	Czechia	6
	Denmark	6
	Dominica	2
	Finland	5
	France	9
	Faroe Islands	[a]
	Germany	6
	Greece	5
	Grenada	0
	Hungary	9
	Ireland	7
	Iceland	3
	Israel	5
	Italy	6
	Jamaica	4
	Jordan	5
	Liechtenstein	[a]

	Luxembourg	5
	Netherlands	6
	North America	3
	Monaco	0
	New Zealand including Cook Islands	8
	Norway	7
	Puerto Rico	1
	San Marino	0
	Saint Lucia	4
	Switzerland	6
	Slovak Republic	7
	Slovenia	8
	Saint Vincent and the Grenadines	5
	Sweden	8
	United Arab Emirates	1
	United States	3
	Virgin Islands, US	4[c]
	West Bank and Gaza	1

IR: incidence rate. TB: tuberculosis.

a: no data, estimated TB incidence by authors

b: Xhevat Kurhasani, Hasan Hafizi, Incidence and case-notification rate of tuberculosis in Kosovo for the period 2000-2010.

c: 2020: https://www.cdc.gov/tb/statistics/reports/2021/table34.htm

Number are rounded an TB IR of 9.8 will therefore appear as 10 in the <10/100.000 group

Data source: The World Health Organization https://data.worldbank.org/indicator/SH.TBS.INCD?locations=1W&most_recent_year_desc=true;

Table S4. Interferon Gamma Release Assay (IGRA) tests in patients with inflammatory bowel or rheumatic disease distributed by diagnosis coding Danish regions, 2010-2018

Region	Persons with IBD or IRD					Background population mid period, 2014		IGRA tested persons of background population
	Persons without IGRA test		IGRA tested persons		Total			
	n	%	n	%	n	n	%	%
North Denmark Region	4,819	72.0	1,877	28.0	6,696	581,057	10.3	0.32
Central Denmark Region	6,531	61.4	4,106	38.6	10,637	1,277,538	22.7	0.32
Region of Southern Denmark	9,274	75.1	3,076	24.9	12,350	1,202,509	21.4	0.26
Capital region of Denmark	12,910	72.5	4,903	27.5	17,813	1,749,405	31.1	0.28
Region Zealand	6,187	76.9	1,854	23.1	8,041	816,726	14.5	0.23
**Total**	**39,721**	**71.5**	**15,816**	**22.1**	**55,537**	**5,627,235**	**100.0**	**0.28**

IBD: Inflammatory bowel disease. IGRA: Interferon Gamma Release Assay. IRD: Inflammatory rheumatic disease.
